# Predictors of Covid-19 level of concern among older adults from the health and retirement study

**DOI:** 10.1038/s41598-022-08332-8

**Published:** 2022-03-15

**Authors:** Hind A. Beydoun, May A. Beydoun, Jordan Weiss, Rana S. Gautam, Sharmin Hossain, Brook T. Alemu, Alan B. Zonderman

**Affiliations:** 1 Department of Research Programs, Fort Belvoir Community Hospital, 9300 DeWitt Loop, Fort Belvoir, VA 22060 USA; 2 Laboratory of Epidemiology and Population Sciences, National Institute on Aging, NIA/NIH/IRP, Baltimore, MD 21225 USA; 3 Department of Demography, UC Berkeley, Berkeley, CA 94720 USA; 4 Department of Sociology and Human Services, University of North Georgia, Dahlonega, GA 30597 USA; 5 Health Sciences Program, School of Health Sciences, Western Carolina University, Cullowhee, NC 28723 USA

**Keywords:** Infectious diseases, Diseases, Risk factors, Signs and symptoms, Mathematics and computing

## Abstract

The purpose of this longitudinal study is to construct a prediction model for Covid-19 level of concern using established Covid-19 socio-demographic, lifestyle and health risk characteristics and to examine specific contributions of obesity-related cardiometabolic health characteristics as predictors of Covid-19 level of concern among a representative sample of U.S. older adults. We performed secondary analyses of existing data on 2872 2006–2020 Health and Retirement Study participants and examined 19 characteristics in relation to the outcome of interest using logistic regression and machine learning algorithms. In mixed-effects ordinal logistic regression models, a history of diabetes, stroke as well as 1–2 cardiometabolic risk factors and/or chronic conditions were associated with greater Covid-19 level of concern, after controlling for confounders. Female sex, birth cohort, minority race, Hispanic ethnicity and total wealth as well as depressive symptoms were associated with higher level of Covid-19 concern, and education was associated with lower level of Covid-19 concern in fully adjusted mixed-effects ordinal logistic regression models. The selected socio-demographic, lifestyle and health characteristics accounted for < 70% of the variability in Covid-19 level of concern based on machine learning algorithms. Independent risk factors for Covid-19 level of concern among U.S. older adults include socio-demographic characteristics and depressive symptoms. Advanced research is needed to identify relevant predictors and elucidate underlying mechanisms of observed relationships.

## Introduction

The coronavirus disease 2019 (Covid-19) caused by the Severe Acute Respiratory Syndrome Coronavirus 2 (SARS-CoV-2) remains a public health concern worldwide and especially in the United States^1–10^. Although a substantial proportion of individuals infected with SARS-CoV-2 may be asymptomatic, symptomatic Covid-19 cases may exhibit a broad spectrum of clinical manifestations and severity^3,8,11–13^. Furthermore, evidence indicates that both asymptomatic and symptomatic Covid-19 cases may transmit the SARS-CoV-2 infection to others^8,11–14^. A wide range of Covid-19 related respiratory and non-respiratory (systemic) symptoms have been reported, the most frequent being fever, dry cough, pneumonia, breathing difficulties (dyspnea), headache, asthenia, anosmia and diarrhea^8,10,13,14^. Covid-19 cases may progress to multi-organ failure^3,6,11,15^ with prognosis often measured by increased need for hospitalization^1,3,6,9,11,16–19^, mechanical ventilation^3,5,9,16^ and/or admission to an intensive care unit (ICU)^3,5,15,16,19,20^ as well as increased risk of death^1–7,10–13,16–22^. There is also growing evidence that Covid-19 may be associated with long-term damage to the brain, heart, kidneys, and other organs as well as a “long-haul” syndrome which may not resolve^23–29^.

Despite continued efforts at flattening the epidemic curve and the gradual emergence of potentially effective Covid-19 treatments and vaccines^1^, as of April 2021, a total of 131 million cases and 2.85 million deaths have been confirmed as attributed to Covid-19 in > 200 countries and territories, worldwide^3,7^. Similarly, 30.8 million confirmed Covid-19 cases and 555,000 Covid-19 deaths have been reported in the United States as of April 2021^2,3,19^. Early in the course of the pandemic, several host characteristics have been identified as SARS-CoV-2 susceptibility and/or Covid-19 prognostic factors thereby contributing to risk stratification and better understanding of the underlying biological mechanisms for Covid-19 onset, progression and outcome^1,3–5,7–10,19,21,30^. Evidence from observational studies have identified specific socio-demographic ^1,3,4,6–9,11–14,17,19,30–33^ (e.g. male sex, advanced age, minority race/ethnicity), lifestyle^4,30,34^ (e.g. smoking) and health^1–3,5,6,10,12,14,17,19–21,30,34,35^ (e.g. body mass index, elevated inflammatory markers, pre-existing chronic conditions such as coronary heart disease, cerebrovascular disease, heart failure, arrhythmias, hypertension, dyslipidemia, diabetes, chronic obstructive pulmonary disease, chronic kidney disease, cancer and multi-morbidity) characteristics as key determinants of Covid-19 infection, clinical manifestation and outcome.

Whereas the disproportionate burden of Covid-19 among men and racial and ethnic minorities and individuals with pre-existing chronic conditions remain an active area of research, immune senescence may be responsible for the over-representation of older people among severe cases of Covid-19 as well as Covid-19 related deaths^2,3,17,30,33^. A highly publicized research finding that has been confirmed by recently conducted meta-analyses^7,10,19^ is that obesity and its associated cardiometabolic risk factors and/or chronic conditions are key determinants of Covid-19 outcome. This finding has been attributed to multiple biological mechanisms including over-expression of angiotensin-converting enzyme 2 (ACE2)^6,11,20^ and excessive inflammatory response^20^ by the adipose tissue.

Due the recent nature of the Covid-19 pandemic, a gap in the literature exists regarding Covid-19 epidemiology awareness, perception of Covid-19 risk and Covid-19 level of concern, especially among older adults who are considered a high-risk group for the detrimental health effects of Covid-19. It remains unclear whether socio-demographic, lifestyle and health characteristics that have been identified in the literature as predictors of Covid-19 risk have become common knowledge and can similarly predict level of concern with Covid-19 among older adults. The level of concern with Covid-19 may reflect an individual’s experience with the pandemic in terms of its psychological burden, risk preference, health literacy or a combination of these characteristics. We hypothesize that an older adult’s profile as it pertains to Covid-19 pathology, severity and risk/protective factors will likely influence their level of concern with the Covid-19 pandemic, which can mitigate their psychological well-being and/or lead to behavioral change such as taking additional precautions to avoid exposure to SARS-CoV-2. Furthermore, the specific contribution of cardiometabolic risk factors and/or chronic conditions to Covid-19 level of concern among older adults remains unknown. The purpose of this longitudinal study is to construct a prediction model for Covid-19 level of concern using established socio-demographic, lifestyle and health-related Covid-19 risk factors and to examine the specific contribution of obesity-related cardiometabolic risk factors and/or chronic conditions as predictors of Covid-19 level of concern among a representative sample of U.S. older adults who participated in the 2006–2020 Health and Retirement Study (HRS).

## Methods

### Data source

Initiated in 1992, the HRS is an ongoing, nationally representative longitudinal study of community-dwelling U.S. adults over the age of 50 and their spouses of any age. The HRS was designed to study economic well-being, labor force participation, health and family composition among older adults through biennial surveys administered by telephone or face-to-face interviews. Although the HRS only interviews community-dwelling adults in their baseline surveys, respondents who enter long-term care facilities are also retained. Multistage probability sampling of U.S. households within geographical strata was performed whereby African Americans, Hispanics and residents of Florida were over-sampled. Response rates at baseline and follow-up waves were > 80% for all HRS interviews. Written informed consent was provided by all participants and the University of Michigan’s Institutional Review Board approved study protocols. The HRS is sponsored by the National Institute on Aging (grant number U01AG009740) and the Social Security Administration. Details of HRS procedures were reported elsewhere^36,37^ with updated documentation and data products available at About | Health and Retirement Study (umich.edu).

### Study participants

The original HRS study consists of participants from whom data were collected in 1992, 1994 and 1996, and the Study of Asset and Health Dynamics of the Oldest Old (AHEAD) consists of those from whom data were collected in 1993 and 1995^38,39^. The two studies were merged and two new generations (the Children of the Depression and the War Babies) were added in 1998. Subsequently, Early Baby Boomers were added in 2004, Mid Baby Boomers were added in 2010 and Late Baby Boomers were added in 2016^38,39^. Starting in 2006, half of the sample completed detailed in-person interviews that included physical, biological and psychosocial measures, and the other half completed a core interview by telephone^38,39^. To reduce study-related costs and burden on participants, enhanced interviewing alternated among half-samples at each subsequent wave^38,39^. This sample was restricted to HRS participants for whom data were collected during 2006, 2008, 2010, 2012, 2014, 2016 and/or 2018 (before Covid-19) waves as well as the 2020 (Covid-19) wave whereby enhanced interviewing was conducted by telephone due to social distancing restrictions^38^. Release to fieldwork occurred sequentially on June 11, 2020 and September 24, 2020 for the 2020 HRS Covid-19 project^38^. To achieve study goals and generate the final study sample, we linked the latest release of the 2020 HRS Covid-19 project, which became publicly available in February 2021 for 3266 respondents, to the 1992–2018 RAND Center for the Study of Aging HRS longitudinal file^38^. Information regarding the 2020 HRS Covid-19 Project can be accessed at 2020 HRS COVID-19 Project | Health and Retirement Study (umich.edu).

### Study variables

The 2006–2020 HRS core data is comprised of standard questionnaire sections that include questionnaire items of interest. In addition, the Covid-19 related questionnaire items were asked during the 2020 HRS wave. As such, we examined a wide range of socio-demographic, lifestyle and health characteristics, at the latest available 2006–2020 HRS wave as well as at multiple HRS waves taking into consideration repeated measurements. Specifically, we defined 7 characteristics identified in the published literature as predictors of Covid-19 infection, progression and/or outcome as well as 12 characteristics that may confound or modify the hypothesized relationship between these predictors and Covid-19 level of concern.

#### Covid-19 level of concern

Self-reported Covid-19 level of concern is a novel concept that has been introduced to several ongoing cohort studies including the HRS, whereby it was determined at a single time point (2020 HRS wave) using one questionnaire item (“Overall, on a scale from 1 to 10, where one is the least concerned and ten is the most concerned, how concerned are you about the coronavirus pandemic?”) This question was asked of 2020 HRS Covid-19 project respondents on an ordinal scale ranging between 1 and 10 and further categorized based on quantiles (‘Low’ [1st tertile], ‘Medium’ [2nd tertile] and ‘High’ [3rd tertile]). We subsequently considered the ‘Medium’ category as a referent or performed pairwise comparisons among these categories depending on the analysis conducted.

#### Socio-demographic characteristics

2006–2020 HRS data were extracted on sex (male, female), birth cohort (Original/AHEAD/Children of the Depression, War Babies, Early Baby Boomers, Mid Baby Boomers, Late Baby Boomers), age (continuous; 50–54, 55–59, 60–64, 65–69, 70–74, 75–79, 80+ years), race (White/Caucasian, Black/African American, Other), ethnicity (Hispanic, non-Hispanic), marital status (never married, married/partnered, separated/divorced, widowed), education (no degree, GED, high school graduate, some college, college degree or higher), work status (working, not working), federal insurance coverage (Yes, No), total wealth (in U.S. dollars) (< 25,000, 25,000–124,999, 125,000–299,999, ≥ 300,000), number of household members (≤ 3, > 3) and Census region of residence (Northeast, Midwest, South, West, Other)^40^. Of these characteristics, sex, age and race/ethnicity are established risk factors for Covid-19. Furthermore, marital status, number of household members and region of residence were available with minimal amount of missing data until 2018, whereas other socio-demographic variables were available until 2020.

#### Lifestyle characteristics

2006–2020 HRS data were extracted on smoking status (never smoker, past smoker, current smoker), frequency of alcohol consumption (abstinent, 1–3 days per month, 1–2 days per week, ≥ 3 days per week) and frequency of moderate/vigorous exercise (never, 1–4 times per month, > 1 time per week). Of these characteristics, smoking^31^ and sedentary lifestyle^32,41^ are established Covid-19 risk factors.

#### Health characteristics

Self-rated health from the 2006–2020 HRS waves was evaluated using a single item (‘‘would you say your health is excellent, very good, good, fair, or poor?’’) and dichotomized as ‘excellent/very good/good’ and ‘fair/poor’. Symptoms of depression were assessed between 2006 and 2018 using a modified 8-item Center for Epidemiological Studies Depression Scale (CES-D) and total CES-D score was calculated with higher scores indicating worse symptoms of depression^37,42^. Self-reported weight and height as well as presence of obesity-related cardiometabolic risk factors and/or chronic conditions were extracted from the 2006–2020 HRS waves of data. Body mass index (BMI) was defined as weight (in kilograms) divided by height (in meters) squared, and categorized as < 25, 25–29.9, ≥ 30 kg/m^2^. The presence of obesity-related cardiometabolic risk factors and/or chronic conditions was determined using a series of standard questions focused on physician-diagnosed hypertension, diabetes, heart disease (heart attack, coronary heart disease, angina, congestive heart failure and/or other heart problems) and stroke. We further categorized the number of obesity-related cardiometabolic risk factors and/or chronic conditions as ‘0’, ‘1–2’ and ‘≥ 3’^37,42^. Neither self-rated health nor symptoms of depression are established Covid-19 risk factors although both of these characteristics are linked to psychological health and risk perception, with self-rated health serving as a proxy of health status^43^. By contrast, BMI as well as obesity-related cardiometabolic risk factors and/or chronic conditions are established risk factors for Covid-19.

### Statistical analysis

Complete subject analyses were conducted using Stata release 16 (StataCorp19. Stata Statistical Software; Release 16. College Station, TX, USA: StataCorp LLC) and R release 4.0.2 (R Core Team (2020). R: A language and environment for statistical computing. R Foundation for Statistical Computing, Vienna, Austria. URL https://www.R-project.org/) while taking into account complex sampling design and using the preliminary survey weight variable for the 2020 HRS Covid-19 sample (*CVWGTR*). Whereas categorical data were summarized using frequencies and percentages, continuous data were summarized by calculating measures of central tendency (mean, median) and dispersion (standard error (SEM), interquartile range), as appropriate. Furthermore, we examined bivariate associations using uncorrected Chi-square and design-based F-tests. We also performed predictive modeling using traditional regression and machine learning (ML) techniques. First, we examined the bivariate association of Covid-19 level of concern with socio-demographic, lifestyle and health characteristics at the latest available HRS wave of data, using multinomial logistic regression modeling, with ‘Medium’ level of Covid-19 concern selected as the referent. Second, we examined the relationship of each cardiometabolic risk factor or chronic condition with Covid-19 level of concern using ordinal and mixed-effects ordinal logistic regression models, while sequentially controlling for socio-demographic, lifestyle and health characteristics. Third, we screened out socio-demographic, lifestyle and health characteristics if they were not related to Covid-19 level of concern in bivariate analyses at α = 0.2, and the remaining variables were entered into ordinal and mixed-effects ordinal logistic regression models. The proportional odds assumption was evaluated prior to using ordinal and mixed-effects ordinal logistic regression. Finally, we applied ML algorithms to select the best predictive model for Covid-19 level of concern defined as a series of dichotomous variables (‘Low’ vs. ‘Medium’, ‘High’ vs. ‘Low’)^44–52^. ML algorithms are more flexible than traditional regression techniques since they can handle a large number of predictors as well as evaluate non-linear relationships and interaction effects, resulting in superior predictive performance. Super Learner is an Ensemble ML algorithm that estimates the performance of an initial set of candidate models called “learners” and creates an optimal weighted average of these models known as a convex combination of algorithms or “Ensemble” using specific performance criterion (e.g. cross-validated area under the receiving operating characteristic curve (cv-AUROC))^44–46,48–54^. The purpose of using Super Learner is to combine the results of multiple parametric and non-parametric models and to evaluate the extent to which socio-demographic, lifestyle and health characteristics are sufficient for predicting Covid-19 level of concern. This algorithm relies on user-defined ML (e.g. Least Absolute Shrinkage and Selection Operator [LASSO], Random Forests, XGBoost, Support Vector Machines [SVM]) algorithms, a V-fold “inner” cross-validation process, a U-fold “outer” cross-validation process and a loss function to identify the best weighted combination of prediction models from multiple candidates based on calibration, discrimination, and risk classification criteria^44–54^. As previously described^44–54^, we selected the majority of HRS participants (80%) as a training sample and 20% of HRS participants as a test sample. Two-sided statistical tests were performed while assuming α = 0.05.

### Ethical approval

Since the project was determined to be research not involving human subjects, a waiver of institutional review board approval was granted at Fort Belvoir Community Hospital. Due to the nature of the research study, informed consent was not needed as determined at Fort Belvoir Community Hospital. The project adhered to relevant ethical guidelines/regulations in accordance with the Declaration of Helsinki.

## Results

**Figure 1:**
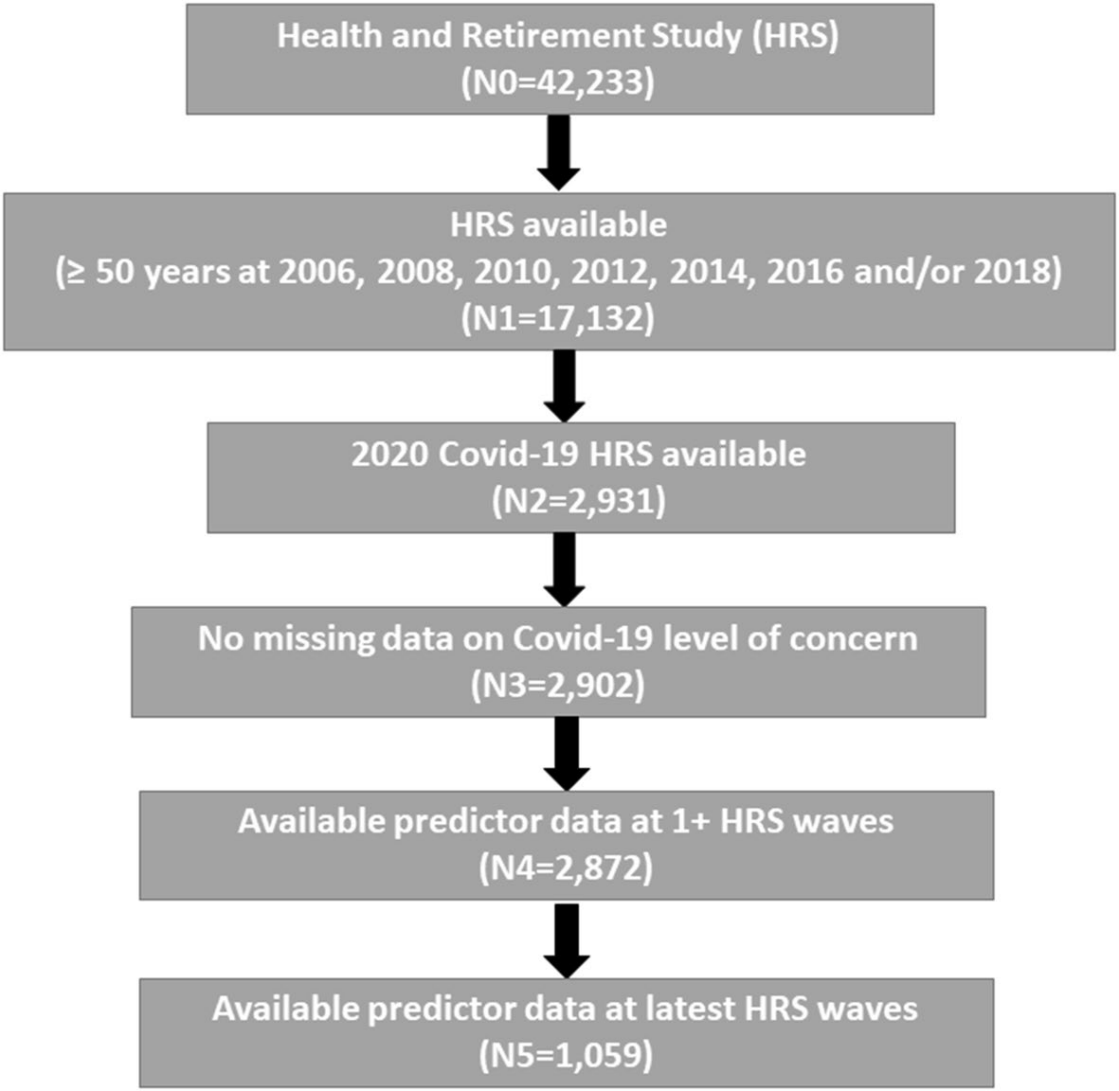
Study flowchart—2006–2020 health and retirement study.

As shown in Fig. 1, 17,132 out of 42,233 HRS participants were ≥ 50 years of age at the 2006, 2008, 2010, 2012, 2014, 2016 or 2018 waves of data. Of those, 2931 HRS participants took part in the 2020 Covid-19 project. Whereas 2902 of HRS participants had data on Covid-19 level of concern, 2872 remained for longitudinal analyses after restricting to those with predictor variables from at least one of the 2006–2020 HRS waves and 1059 remained for cross-sectional analyses after excluding those with missing predictor variables at the 2020 HRS wave. Accordingly, 2872 HRS participants were used for longitudinal (mixed-effects) analyses and 1059 HRS participants were used for cross-sectional analyses involving data from the 2020 Covid-19 project alone.

Table S.1 presents socio-demographic, lifestyle and health characteristics at the latest wave of data according to Covid-19 level of concern in which we selected ‘Medium’ level of Covid-19 concern as a referent. The analytic sample consists of 1059 HRS participants of whom 227 reported ‘Medium’ (8–9) level of concern (referent), whereas 315 reported ‘Low’ (1–7) level of concern, and 517 reported ‘High’ (10) level of concern with the Covid-19 pandemic. Compared to HRS participants who elicited ‘Medium’ level of Covid-19 concern, those with ‘Low’ level of Covid-19 concern had significantly more current smokers versus never smokers (Relative Risk Ratio (RRR) = 3.66, 95% Confidence Interval (CI) 1.61, 8.30), with fewer women versus men (RRR = 0.54, 95% CI 0.34, 0.89), War Babies versus Original/AHEAD/Children of the Depression (RRR = 0.48, 95% CI 0.24, 0.93), Black/African Americans versus White/Caucasians (RRR = 0.45, 95% CI 0.23, 0.88) and individuals with a total wealth ranging between $125,000 and $299,999 versus < $25,000 (RRR = 0.41, 95% CI 0.18, 0.96). Compared to HRS participants eliciting a ‘Medium’ level of Covid-19 concern, those with ‘High’ level of Covid-19 concern had significantly more Black/African Americans versus White/Caucasians (RRR = 2.70, 95% CI 1.59, 4.56), current smokers versus never smokers (RRR = 2.39, 95% CI 1.12, 5.14), individuals with fair or poor self-rated health versus excellent, very good or good self-rated health (RRR = 1.63, 95% CI 1.03, 2.58) and significantly fewer individuals who were high school versus no degree (RRR = 0.45, 95% CI 0.23, 0.86) or college graduates versus no degree (RRR = 0.23, 95% CI 0.12, 0.46), those with a total wealth ranging between $25,000 and $299,999 versus < $25,000 and those who consumed alcohol ≥ 3 days per week versus abstainers (RRR = 0.51, 95% CI 0.28, 0.92). There was significant positive association of ‘High’ versus ‘Medium’ level of Covid-19 concern with the depression symptoms score (RRR = 1.15, 95% CI 1.03, 1.29) as well as history of hypertension (RRR = 1.72, 95% CI 1.08, 2.73).

Table 1 displays findings from a set of sequentially adjusted ordinal and mixed-effect ordinal logistic regression models to examine obesity-related cardiometabolic risk factors and/or chronic conditions in relation to Covid-19 level of concern. Hypertension was associated with greater level of Covid-19 concern only in unadjusted models (OR = 1.43, 95% CI 1.04, 1.97 [ordinal logistic regression]; OR = 1.26, 95%CI 1.16, 1.36 [mixed-effects ordinal logistic regression]). Upon sequential adjustment for socio-demographic, lifestyle and health characteristics, these findings became statistically non-significant. By contrast, diabetes was associated with greater level of Covid-19 concern in the unadjusted (OR = 1.19, 95% CI 1.08, 1.32) and fully-adjusted (OR = 1.18, 95% CI 1.05, 1.33) mixed-effects logistic regression models. In fully-adjusted mixed effects ordinal logistic regression models, a history of stroke (OR = 1.28, 95% CI 1.03, 1.58) and having ‘1–2’ versus ‘0’ cardio-metabolic risk factors and/or chronic conditions (OR = 1.13, 95% CI 1.01, 1.26) were significantly associated with level of Covid-19 concern.

**Table 1:** Ordinal logistic regression and mixed effects ordinal logistic regression models for each obesity-related cardiometabolic risk factor and chronic condition as a predictor of Covid-19 level of concern before and after controlling for socio-demographic, lifestyle and health characteristics—2020 Health and Retirement Study enhanced interviewing Covid-19 half-sample.

	Model I^a^		Model II^b^		Model III^c^		Model IV^d^	
	OR	95% CI	OR	95% CI	OR	95% CI	OR	95% CI
**Ordinal**								
Body mass index (continuous)	0.99	(0.99, 1.00)	0.99	(0.98, 1.00)	0.99	(0.98, 1.00)	0.99	(0.98, 1.00)
**Body mass index (categorical)**								
< 25	Ref		Ref		Ref		Ref	
25–29.9	0.96	(0.64, 1.42)	1.14	(0.75, 1.73)	1.16	(0.77, 1.76)	1.06	(0.69, 1.61)
≥ 30	0.89	(0.61, 1.30)	0.96	(0.64, 1.44)	0.99	(0.66, 1.52)	0.97	(0.63, 1.49)
Hypertension	1.43	(1.04, 1.97)	1.16	(0.83, 1.63)	1.21	(0.86, 1.70)	1.08	(0.76, 1.55)
Diabetes	0.98	(0.69, 1.38)	0.91	(0.64, 1.29)	0.93	(0.65, 1.33)	0.87	(0.59, 1.27)
Heart disease	1.22	(0.88, 1.70)	1.19	(0.84, 1.71)	1.19	(0.83, 1.71)	1.04	(0.71, 1.55)
Stroke	0.81	(0.51, 1.30)	0.75	(0.45, 1.23)	0.76	(0.46, 1.26)	0.73	(0.43, 1.22)
**Number of cardiometabolic risk factors and chronic conditions**								
0	Ref		Ref		Ref		Ref	
1–2	1.24	(0.85, 1.79)	0.96	(0.64, 1.42)	0.99	(0.66, 1.48)	0.93	(0.61, 1.42)
≥ 3	1.26	(0.76, 2.08)	0.95	(0.55, 1.65)	0.96	(0.56, 1.66)	0.76	(0.43, 1.33)
**Mixed effects ordinal**								
Body mass index (continuous)	1.00	(0.99, 1.01)	0.99	(0.99, 1.00)	1.00	(0.99, 1.01)	1.00	(0.99, 1.01)
**Body mass index (categorical)**								
< 25	Ref		Ref		Ref		Ref	
25–29.9	1.06	(0.95, 1.17)	1.12	(1.00, 1.26)	1.14	(1.01, 1.29)	1.12	(0.99, 1.26)
≥ 30	1.09	(0.98, 1.20)	1.06	(0.94, 1.19)	1.12	(0.99, 1.27)	1.10	(0.98, 1.25)
Hypertension	1.26	(1.16, 1.36)	1.05	(0.95, 1.16)	1.06	(0.96, 1.17)	1.05	(0.95, 1.16)
Diabetes	1.19	(1.08, 1.32)	1.14	(1.02, 1.27)	1.19	(1.06, 1.33)	1.18	(1.05, 1.33)
Heart disease	1.04	(0.94, 1.15)	1.04	(0.92, 1.17)	1.07	(0.95, 1.22)	1.03	(0.91, 1.17)
Stroke	1.06	(0.88, 1.27)	1.19	(0.97, 1.46)	1.29	(1.04, 1.59)	1.28	(1.03, 1.58)
**Number of cardiometabolic risk factors and chronic conditions**								
0	Ref		Ref		Ref		Ref	
1–2	1.29	(1.19, 1.41)	1.11	(0.99, 1.23)	1.14	(1.02, 1.27)	1.13	(1.01, 1.26)
≥ 3	1.24	(1.05, 1.46)	1.18	(0.97, 1.82)	1.24	(1.01, 1.50)	1.18	(0.96, 1.44)

^a^Model I is unadjusted.

^b^Model II is adjusted for socio-demographic characteristics.

^c^Model III is adjusted for socio-demographic and lifestyle characteristics.

^d^Model IV is adjusted for socio-demographic, lifestyle and health characteristics.

Table 2 shows ordinal logistic and mixed-effects ordinal logistic regression models whereby key socio-demographic, lifestyle and health characteristics identified in the bivariate analysis at α = 0.20 were entered into fully-adjusted models for Covid-19 level of concern. Female sex, older age, minority race (Black/African American versus White/Caucasian, Other versus White/Caucasian), ethnicity (Hispanic versus Non-Hispanic), total wealth (‘≥ $25,000’ versus ‘< $25,000’) and higher depressive symptoms score were key predictors for higher level of Covid-19 concern, whereas education (‘College degree or higher’ versus ‘No degree’) and birth cohort (‘Mid or Late Baby Boomers’ versus ‘Original/AHEAD/Children of the Depression’) were associated with lower level of Covid-19 concern in at least one of these two models.

**Table 2:** Ordinal logistic regression and mixed effects ordinal logistic regression models for major predictors of Covid-19 level of concern—2020 Health and Retirement Study enhanced interviewing Covid-19 half-sample.

	Model I^a^		Model II^b^	
	OR	95% CI	OR	95% CI
**Sex**				
Male	Ref			
Female	1.81	(1.27, 2.59)	1.65	(1.49, 1.83)
Age (years)	1.07	(1.00, 1.13)	1.01	(0.99, 1.02)
**Birth cohort**				
Original/AHEAD/children of the depression	Ref			Ref
War Babies	1.96	(0.96, 3.97)	0.88	(0.76, 1.02)
Early baby boomers	2.38	(0.85, 6.71)	0.88	(0.73, 1.08)
Mid baby boomers	2.84	(0.77, 10.47)	0.75	(0.59, 0.95)
Late baby boomers	3.95	(0.78, 20.0)	0.73	(0.55, 0.97)
**Race**				
White/Caucasian	Ref			Ref
Black/African American	4.80	(3.05, 7.55)	4.08	(3.46, 4.81)
Other	1.30	(0.70, 2.42)	1.24	(1.03, 1.50)
**Ethnicity**				
Hispanic	2.00	(1.17, 3.42)	2.49	(2.06, 3.02)
Non-Hispanic	Ref			Ref
**Education**				
No degree	Ref			Ref
GED	0.86	(0.38, 1.94)	0.93	(0.72, 1.21)
High school graduate	0.71	(0.41, 1.22)	0.84	(0.69, 1.03)
Some college	0.78	(0.44, 1.35)	0.83	(0.67, 1.01)
College degree or higher	0.49	(0.27, 0.91)	0.77	(0.62, 0.94)
**Work status**				
Working	0.98	(0.62, 1.57)	1.00	(0.89, 1.12)
Not working	Ref			Ref
**Federal health insurance coverage**				
Yes	1.67	(0.98, 2.87)	0.96	(0.83, 1.11)
No	Ref			Ref
**Total wealth ($)**				
< 25,000	Ref			Ref
25,000–124,999	1.00	(0.68, 1.48)	1.27	(1.12, 1.45)
125,000–299,999	1.21	(0.65, 2.25)	1.35	(1.13, 1.61)
≥ 300,000	1.89	(0.59, 6.07)	1.29	(1.01, 1.65)
**Smoking status**				
Never smoker	Ref			Ref
Past smoker	0.90	(0.63, 1.29)	1.08	(0.98, 1.19)
Current smoker	0.60	(0.33, 1.09)	0.97	(0.82, 1.14)
**Frequency of alcohol consumption**				
Abstinent	Ref			Ref
1–3 days per month	0.99	(0.62, 1.61)	0.89	(0.79, 1.02)
1–2 days per week	0.93	(0.60, 1.43)	1.01	(0.88, 1.15)
≥ 3 days per week	0.98	(0.58, 1.63)	1.00	(0.86, 1.16)
**Self-rated health**				
Excellent/very good/good	Ref			Ref
Fair/poor	1.03	(0.71, 1.48)	0.88	(0.76, 1.03)
**Depression symptoms score**	1.10	(1.00, 1.22)	1.10	(1.07, 1.14)
**Body mass index (kg/m**^**2**^**)**	0.99	(0.98, 1.00)	0.99	(0.99, 1.00)
**Hypertension**				
Yes	1.06	(0.74, 1.51)		1.02 (0.92, 1.14)
No	Ref			Ref

*AHEAD* study of asset and health dynamics of the oldest old, *GED* general educational development.

^a^Ordinal logistic regression model.

^b^Mixed effects logistic regression model.

Table S.2 presents the outcome of the Super Learner model for predictors of Covid-19 level of concern, using four distinct ML algorithms. Whereas ‘Random Forest’ had the lowest cv-Risk suggesting less error in predicting Covid-19 level of concern, ‘XGBoost’ had the highest cv-Risk suggesting more error in predicting Covid-19 level of concern. The weighted average cv-Risk for the Super Learner model was 0.247 for ‘Low’ versus ‘Medium’ level of Covid-19 concern with an cv-AUROC = 0.485. By contrast, the weighted average cv-Risk for the Super Learner model was 0.213 for ‘High’ versus ‘Low’ level of concern with an cv-AUROC = 0.664. Accordingly, the Super Learner model was somewhat predictive of ‘High’ versus ‘Low’ but did not perform beyond a chance finding for comparing ‘Low’ versus ‘Medium’ level of concern. The cv-AUROC suggest that < 70% of the variability in COVID-19 level of concern is explained by the selected variables.

## Discussion

In this longitudinal study involving HRS participants, we evaluated socio-demographic, lifestyle and health characteristics as predictors of Covid-19 level of concern, while focusing on the potential role played by obesity-related cardiometabolic risk factors and/or chronic conditions among older adults, a high-risk group for Covid-19. Study results suggested that history of diabetes, stroke as well as 1–2 cardiometabolic risk factors and/or chronic conditions were significantly related to Covid-19 level of concern after adjustment for confounders. By contrast, female sex, older age, minority race, Hispanic ethnicity, total wealth and higher depressive symptoms score were associated with higher level of Covid-19 concern, and education was associated with lower level of Covid-19 concern in fully adjusted ordinal logistic regression models. Super Learner models for predictors of Covid-19 level of concern resulted in cv-AUROC that did not exceed 0.7.

Although numerous systematic reviews and meta-analyses have established obesity and its associated cardiometabolic risk factors and chronic conditions as predictors of Covid-19 infection and/or prognosis^7,10,19^, study results indicate that few of these cardiometabolic health characteristics were independent predictors of Covid-19 level of concern after controlling for confounders. As suggested by Ponsford et al. and Rodilla et al. a cause-and-effect relationship between cardiometabolic health and Covid-19 characteristics may not be easily established using observational study designs, and associations could potentially be confounded by stronger risk factors such as age^9,34^. Regardless, a recent review on possible links to Covid-19 implies that adipose tissue might serve as a pathogen reservoir, accelerating transmission of the virus in people with underlying comorbid conditions such as obesity. Diabetes was implicated as a result of increased inflammatory response by C type leptin receptors (present in adipose tissues), while increased ACE2 expression was identified as an entryway for the virus among hypertensive individuals^20^. The latter is particularly of concern because ACE2 expression is one of the highest in adipose tissue and individuals with lower ACE2 expression were found to be less susceptible to Covid-19^55^. Uncontrolled inflammatory response to any triggers has been a key driver in propagating the virus in the system, which was exacerbated by additional underlying conditions such as obesity, type 2 diabetes and hypertension which tend to co-occur. The absence of an association between Covid-19 level of concern with having three or more cardiometabolic health characteristics is likely due to sample size limitations. Moreover, the preponderance of socio-demographic characteristics as predictors suggests that disparities in Covid-19 level of concern may be the outcome of inadequate knowledge of the Covid-19 epidemiology, including host characteristics that may impact Covid-19 risk.

On the other hand, this study highlights the importance of demographic (sex, age, race, ethnicity), socioeconomic (education, total wealth) and health (depressive symptoms) features as key predictors of Covid-19 level of concern. Because aging has been associated with immune senescence and with a greater host susceptibility to infectious disease, in general, and detrimental health effects of Covid-19, in particular, an increasing level of Covid-19 concern with age is expected assuming awareness among study participants that advanced age is an established Covid-19 risk factor^3,9,11,12,14,30,33,56^. Although previous studies have suggested that Covid-19 disproportionately affects men, the Centers for Disease Control and Prevention has attributed sex differences in Covid-19 infection, progression and/or outcome to a wide range of social, behavioral and psychological characteristics that distinguish men and women^1,4,30^. In particular, evidence suggests that men were more likely to be smokers and less likely to adopt preventive strategies such as face mask-wearing or to initiate and comply with Covid-19 treatments^1,4,12,14,19^. The finding that women were more concerned about the Covid-19 pandemic is, therefore, consistent with previously identified sex differences leading to lower susceptibility to Covid-19 and/or better prognosis after Covid-19 infection among women. Interestingly, lifestyle factors such as poor sleep, physical inactivity and time spent indoors are all believed to compound an already aggravated inflammatory response from the infection, regardless of an individual’s sex, the common link being high stress levels and increased production of pro-inflammatory cytokines, according to recent studies^32,35^. Moreover, pre-existing poor nutritional status might have been an additional, severely understudied risk factor in the Covid-19 literature but highly relevant due to direct links between diet and inflammation^16,56^. This is particularly true for vitamin D status which has the potential for being a novel Covid-19 therapeutic target^15^ irrespective of age, sex and race.

The finding that specific racial and ethnic minorities were more concerned than other groups about the Covid-19 pandemic may be explained by their greater risk of exposure to SARS-CoV-2 as well as the greater impact of the Covid-19 pandemic on their daily lives. Although the same principle can be applied to individuals of lower socioeconomic standing, results pertaining to level of education and total wealth were less clear-cut. Previously conducted studies have implied that the Covid-19 pandemic may have predominantly affected racial and ethnic minorities^1–3,12,17,31,35^ and individuals of low socioeconomic status^3,17,35^. Highton et al. acknowledged that Black and South Asian communities were an especially vulnerable Covid-19 population after controlling for socioeconomic status and cardiometabolic disease, and they were more likely to experience Covid-19 related psychosocial stress and depression^17^. Similarly, Koliaki et al. indicated that beyond sex and age differences, there were racial and ethnic disparities with disproportionate burden of Covid-19 infection and/or mortality affecting non-Hispanic Blacks in the United States^3^. In an attempt to explain sex and minority racial/ethnic background differences in Covid-19 through cardiometabolic, socioeconomic and behavioral factors, Raisi-Estabragh et al. analyzed data from 4510 UK Biobank participants who were tested for Covid-19, of whom 1326 tested positive for Covid-19^31^. Their results suggested that men as well as Black, Asian and Minority Ethnic (BAME) groups were overrepresented among individuals with positive Covid-19 tests^31^. Although the BAME group exhibited poorer cardiometabolic profile, lower 25(OH)-vitamin D, greater material deprivation, and more often lived in larger households and/or flats/apartments, male sex, BAME ethnicity, higher BMI, higher Townsend deprivation score and household overcrowding were independent predictors of Covid-19 positivity^31^. More importantly, cardiometabolic, socio-demographic and behavioral factors did not seem to mediate the relationship of sex or racial/ethnic background with Covid-19 positivity^31^.

The HRS is a large, nationally representative study with > 20 years of longitudinal data covering several cohorts and it includes a wide range of socio-demographic, lifestyle and health-related factors. Nevertheless, study findings need to be interpreted with caution and in light of several limitations. First, the linkage of 2006–2018 HRS with 2020 HRS Covid-19 project data and missing information on key variables yielded analytic samples that were much smaller than the full HRS sample potentially leading to selection bias. Potentially eligible participants and the analytic samples were comparable on distribution by sex, birth cohort and race but differed according to ethnicity and level of education (Table S.3). Second, the majority of HRS data, including Covid-19 level of concern and its hypothesized predictors were self-reported, potentially leading to non-differential misclassification and measures of association that are biased towards the null value. Given sample size limitations, we relied on core 2006–2020 HRS data to perform our analyses, limiting availability of direct assessment of weight and height to calculate BMI as well as dried blood spot biomarkers to determine cardiometabolic risk. Furthermore, the cut-points defining tertiles for Covid-19 level of concern were empirically defined and have not been previously validated using a larger sample size. Third, data analyses have been conducted using observational HRS data and, as such, the estimated relationships are prone to confounding bias and cannot be deemed causal in nature. Notably, depressive symptoms score is likely a correlate of rather than a risk factor for Covid-19 level of concern. Fourth, this study involves secondary analysis of existing HRS data and topics consistently covered by the 2006–2020 waves of HRS data may or may not have yielded the most relevant predictors of Covid-19 level of concern, as suggested by the Super Learner model. Another limitation is the scarcity of literature on this topic that can guide the choice of regression and ML models. Finally, there are two basic models of human behavior, namely, the rational model in which individuals may base their behavior and feelings on logical evaluations of risks and rewards and the irrational model in which individuals may base their behavior on emotions largely derived from their dispositions, aspirations, and fears. With the exception of depressive symptoms, our study covers the rational basis but does not address the irrational basis underlying Covid-19 level of concern. On the other hand, depressive symptoms predict but are not synonymous with clinical depression. Accordingly, it is important to acknowledge that these analyses do not account for dispositional effects whereby an individual who tends to have psychological problems will nearly always endorse concern with Covid-19.

## Conclusions

Socio-demographic characteristics (sex, age, race, ethnicity, education, total wealth) and depressive symptoms may be useful for predicting Covid-19 level of concern among U.S. older adults, a high-risk population for the detrimental health effects of Covid-19. Although current evidence suggests that men may be more susceptible to adverse outcomes related to Covid-19, women were more concerned about the Covid-19 pandemic. Whereas the relationship of Covid-19 level of concern with socioeconomic factors appears to be complex, belonging to racial/ethnic minority groups and advanced age which are key predictors of Covid-19 susceptibility were also key predictors of Covid-19 level of concern. Covid-19 level of concern may be a marker of Covid-19 level of awareness which could affect health behavior, but it may also be a marker of perceived Covid-19 risk with implications for physical, mental and social health. Socio-demographic, lifestyle and health factors that shape Covid-19 level of concern have implications for understanding a population’s response to public health initiatives aimed at stemming the spread of the virus. Given the limitations of the HRS data and the results of the Super Learner model, further research is needed to identify additional predictors and to elucidate the underlying mechanisms of the observed relationships. Finally, more research is needed to evaluate whether Covid-19 level of concern can mediate the relationship between socio-demographic, lifestyle and health characteristics with behavioral outcomes such as face mask-wearing and uptake of vaccines.

## Supplementary Information

Supplementary Tables.

## Data Availability

The data that support the findings of this study are available from the University of Michigan but restrictions apply to the availability of these data. Specifically, the University of Michigan requires researchers wishing to analyze their HRS data to create an account that allows them to download publicly available datasets. Data are however available from the authors upon reasonable request and with permission of the University of Michigan.
