# Supplementary material for: Predictors of Covid-19 level of concern among older adults from the health and retirement study
Source: Sci Rep. 2022 Mar 15;12:4396. doi: 10.1038/s41598-022-08332-8 (PMC8921703; doi:10.1038/s41598-022-08332-8)
Supplement: Supplementary file 1 — Supplementary Tables. [file 41598_2022_8332_MOESM1_ESM.docx]

**Table S.1.** Socio-demographic, lifestyle and health characteristics at the latest wave of data according to Covid-19 level of concern – 2020 Health and Retirement Study enhanced interviewing Covid-19 half-sample (n=1,059) ^a^

|  | **Total**  **(N=1,059)** | **Covid-19 level of concern** | | | | |
| --- | --- | --- | --- | --- | --- | --- |
|  |  | **Medium**  **(8-9)**  **(N=227)** | **Low**  **(1-7)**  **(N=315)** | | **High**  **(10)**  **(N=517)** | |
|  | **%^b^** | **%^b^** | **%^b^** | **RRR ^c^**  **(95% CI)** | **%^b^** | **RRR ^d^**  **(95% CI)** |
| **SOCIO-DEMOGRAPHIC:** |  |  |  |  |  |  |
| ***Sex:*** | P = 0.002 | | | | | |
| Male | 39.6 | 35.7 | 50.3 | Ref. | 33.5 | Ref. |
| Female | 60.3 | 64.3 | 49.7 | 0.54 (0.34, 0.89) | 60.3 | 1.10 (0.70, 1.74) |
| ***Age (years):*** | P = 0.09 | | | | | |
| Mean ± SEM | 68.4 ± 0.4 | 68.5 ± 0.8 | 67.1 ± 0.7 | 0.98 (0.97, 1.00) | 69.3 ± 0.6 | 1.00 (0.98, 1.02) |
|  | P=0.08 | | | | | |
| 50-54 | 0.7 | 0.2 | 0.2 | Ref. | 1.3 | Ref. |
| 55-59 | 20.8 | 17.6 | 22.9 | 0.83 (0.06, 11.3) | 20.8 | 0.15 (0.01, 2.56) |
| 60-64 | 24.5 | 23.0 | 32.0 | 0.88 (0.06, 12.1) | 19.2 | 0.11 (0.01, 1.81) |
| 65-69 | 13.4 | 16.9 | 10.7 | 0.40 (0.03, 5.6) | 13.5 | 0.11 (0.01, 1.74) |
| 70-74 | 13.3 | 15.8 | 10.3 | 0.42 (0.03, 5.71) | 14.4 | 0.12 (0.01, 1.98) |
| 75-79 | 9.4 | 11.4 | 6.0 | 0.34 (0.02, 4.67) | 10.9 | 0.12 (0.01, 2.08) |
| ≥ 80 | 17.9 | 15.0 | 17.7 | 0.75 (0.06, 9.92) | 19.8 | 0.17 (0.01, 2.80) |
| ***Birth cohort:*** | P=0.11 | | | | | |
| Original/AHEAD  /Children of the Depression | 20.0 | 17.0 | 19.3 | Ref. | 22.3 | Ref. |
| War Babies | 15.3 | 18.9 | 10.3 | 0.48 (0.24, 0.93) | 17.3 | 0.69 (0.39, 1.26) |
| Early Baby Boomers | 12.2 | 14.7 | 9.4 | 0.56 (0.27, 1.17) | 13.0 | 0.68 (0.36, 1.28) |
| Mid Baby Boomers | 24.2 | 23.4 | 28.4 | 1.07 (0.57, 1.98) | 21.3 | 0.69 (0.39, 1.24) |
| Late Baby Boomers | 28.2 | 25.9 | 32.6 | 1.11 (0.58, 2.12) | 26.1 | 0.77 (0.42, 1.41) |
| ***Race:*** | P < 0.0001 | | | | | |
| White/Caucasian | 74.9 | 80.3 | 85.5 | Ref. | 63.5 | Ref. |
| Black/African American | 14.7 | 11.3 | 5.4 | 0.45 (0.23, 0.88) | 24.1 | 2.70 (1.59, 4.56) |
| Other | 10.4 | 8.5 | 9.1 | 1.00 (0.46, 2.21) | 12.5 | 1.86 (0.92, 3.77) |
| ***Ethnicity:*** | P=0.01 | | | | | |
| Hispanic | 10.7 | 9.9 | 6.8 | 0.66 (0.35, 1.28) | 14.3 | 1.52 (0.85, 2.72) |
| Non-Hispanic | 89.3 | 90.1 | 93.2 | Ref. | 85.7 | Ref. |
| ***Education:*** | P = 0.004 | | | | | |
| No degree | 15.8 | 10.3 | 11.9 | Ref. | 22.1 | Ref. |
| GED | 6.4 | 5.3 | 6.7 | 1.11 (0.30, 4.11) | 6.7 | 0.59 (0.19, 1.86) |
| High School graduate | 28.2 | 29.1 | 27.9 | 0.84 (0.39, 1.76) | 27.9 | 0.45 (0.23, 0.86) |
| Some college | 28.8 | 26.9 | 29.6 | 0.96 (0.43, 2.11) | 29.2 | 0.51 (0.25, 1.01) |
| College degree or higher | 20.8 | 28.5 | 23.8 | 0.73 (0.34, 1.57) | 14.0 | 0.23 (0.12, 0.46) |
| ***Marital status:*** |  | P=0.27 | | | | |
| Never married | 10.9 | 9.1 | 9.3 | Ref. | 13.4 | Ref. |
| Married / Partnered | 41.7 | 49.2 | 40.8 | 0.81 (0.30, 2.19) | 38.3 | 0.53 (0.22, 1.27) |
| Separated / Divorced | 26.0 | 23.0 | 30.3 | 1.29 (0.44, 3.72) | 24.4 | 0.72 (0.28, 1.84) |
| Widowed | 21.2 | 18.7 | 19.7 | 1.03 (0.37, 2.91) | 23.9 | 0.86 (0.35, 2.15) |

| ***Work status:*** | P=0.06 | | | | | |
| --- | --- | --- | --- | --- | --- | --- |
| Working | 33.5 | 33.7 | 39.8 | 1.30 (0.77, 2.21) | 28.4 | 0.78 (0.47, 1.28) |
| Not working | 66.5 | 66.3 | 60.2 | Ref. | 71.7 | Ref. |
| ***Federal health insurance coverage:*** | P=0.002 | | | | | |
| Yes | 71.1 | 71.6 | 61.7 | 0.64 (0.36, 1.12) | 78.4 | 1.44 (0.84, 2.46) |
| No | 28.8 | 28.4 | 38.3 | Ref. | 21.6 | Ref. |
| ***Total wealth ($):*** | P=0.004 | | | | | |
| < 25,000 | 39.1 | 31.4 | 34.8 | Ref. | 46.9 | Ref. |
| 25,000–124,999 | 48.4 | 49.2 | 53.4 | 0.98 (0.59, 1.63) | 44.0 | 0.59 (0.38, 0.94) |
| 125,000–299,999 | 9.1 | 17.3 | 7.9 | 0.41 (0.18, 0.96) | 5.5 | 0.21 (0.09, 0.48) |
| ≥ 300,000 | 2.6 | 2.1 | 3.9 | 1.68 (0.29, 9.79) | 3.5 | 1.11 (0.22, 5.54) |
| ***Number of household members:*** | P=0.40 | | | | | |
| Mean ± SEM | 2.1 ± 0.05 | 2.1 ± 0.1 | 1.9 ± 0.1 | 0.95 (0.81, 1.13) | 2.1 ± 0.1 | 1.05 (0.89, 1.23) |
|  | P=0.53 | | | | | |
| ≤ 3 | 88.0 | 88.6 | 89.6 | Ref. | 86.4 | Ref. |
| > 3 | 11.9 | 11.4 | 10.4 | 0.89 (0.42, 1.90) | 13.6 | 1.22 (0.59, 2.49) |
| ***Census region of residence:*** | P=0.80 | | | | | |
| Northeast | 16.7 | 18.2 | 15.8 | Ref. | 16.8 | Ref. |
| Midwest | 23.5 | 24.1 | 26.4 | 1.26 (0.58, 2.77) | 20.9 | 0.94 (0.48, 1.87) |
| South | 39.7 | 35.5 | 38.8 | 1.26 (0.60, 2.54) | 42.8 | 1.31 (0.70, 2.42) |
| West | 19.9 | 22.1 | 18.9 | 0.99 (0.44, 2.24) | 19.6 | 0.96 (0.46, 2.03) |
| **LIFESTYLE:** |  |  |  |  |  |  |
| ***Smoking status:*** | P=0.008 | | | | | |
| Never smoker | 41.1 | 41.8 | 37.5 | Ref. | 43.7 | Ref. |
| Past smoker | 42.5 | 51.4 | 40.3 | 0.87 (0.53, 1.43) | 39.4 | 0.73 (0.47, 1.15) |
| Current smoker | 16.3 | 6.8 | 22.3 | 3.66 (1.61, 8.30) | 16.9 | 2.39 (1.12, 5.14) |
| ***Frequency of alcohol consumption:*** | P=0.15 | | | | | |
| Abstinent | 44.9 | 42.1 | 41.6 | Ref. | 49.2 | Ref. |
| 1-3 days per month | 18.4 | 14.1 | 18.7 | 1.34 (0.67, 2.70) | 20.6 | 1.25 (0.67, 2.34) |
| 1-2 days per week | 21.8 | 24.3 | 23.9 | 1.00 (0.54, 1.85) | 18.7 | 0.66 (0.37, 1.18) |
| ≥3 days per week | 14.8 | 19.6 | 15.7 | 0.81 (0.41, 1.62) | 11.6 | 0.51 (0.28, 0.92) |
| ***Frequency of moderate***  ***/vigorous physical exercise:*** | P=0.37 | | | | | |
| Never | 21.7 | 20.2 | 21.0 | Ref. | 22.9 | Ref. |
| 1-4 times per month | 26.3 | 20.7 | 29.8 | 1.38 (0.71, 2.69) | 26.7 | 1.14 (0.62, 2.07) |
| > 1 times per week | 52.0 | 59.1 | 49.2 | 0.79 (0.44, 1.42) | 52.0 | 0.74 (0.44, 1.26) |
| **HEALTH:** |  |  |  |  |  |  |
| ***Self-rated health:*** | P=0.10 | | | | | |
| Excellent/very good/good | 62.3 | 68.9 | 63.3 | Ref. | 57.7 | Ref. |
| Fair/poor | 37.7 | 31.0 | 36.7 | 1.29 (0.77, 2.16) | 42.3 | 1.63 (1.03, 2.58) |
| ***Depression symptoms score:*** | P = 0.01 | | | | | |
| Mean ± SEM | 2.5 ± 0.1 | 2.3 ± 0.2 | 2.4 ± 0.1 | 1.03 (0.91, 1.17) | 2.8 ± 0.1 | 1.15 (1.03, 1.29) |

| ***Body mass index (kg/m^2^):*** | P=0.16 | | | | | |
| --- | --- | --- | --- | --- | --- | --- |
| Mean ± SEM | 30.3 ± 0.4 | 31.6 ± 1.3 | 30.4 ± 0.6 | 0.99 (0.98, 1.00) | 29.5 ± 0.5 | 0.99 (0.97, 1.00) |
|  | P=0.90 | | | | | |
| <25 | 25.3 | 23.1 | 25.1 | Ref. | 26.8 | Ref. |
| 25-29.9 | 31.4 | 33.8 | 30.2 | 0.82 (0.46, 1.47) | 31.2 | 0.79 (0.47, 1.34) |
| ≥30 | 43.2 | 43.1 | 44.7 | 0.96 (0.55, 1.67) | 42.1 | 0.84 (0.50, 1.40) |
| ***Cardiometabolic risk factors***  ***and chronic conditions:*** |  |  |  |  |  |  |
| *Hypertension:* | P=0.03 | | | | | |
| Yes | 63.2 | 57.1 | 59.7 | 1.11 (0.68, 1.82) | 69.6 | 1.72 (1.08, 2.73) |
| No | 36.7 | 42.9 | 40.3 | Ref. | 30.4 | Ref. |
| *Diabetes:* | P=0.73 | | | | | |
| Yes | 28.0 | 25.5 | 29.3 | 1.21 (0.73, 2.00) | 28.4 | 1.16 (0.74, 1.82) |
| No | 71.9 | 74.5 | 70.7 | Ref. | 71.6 | Ref. |
| *Heart disease:* | P=0.36 | | | | | |
| Yes | 30.2 | 27.6 | 28.2 | 1.03 (0.64, 1.67) | 33.2 | 1.30 (0.84, 2.03) |
| No | 69.8 | 72.5 | 71.8 | Ref. | 66.8 | Ref. |
| *Stroke:* | P=0.64 | | | | | |
| Yes | 10.9 | 10.1 | 12.6 | 1.28 (0.63, 2.62) | 9.9 | 0.99 (0.52, 1.90) |
| No | 89.1 | 89.9 | 87.4 | Ref. | 90.0 | Ref. |
| *Number of conditions:* | P=0.56 | | | | | |
| 0 | 25.2 | 30.2 | 26.2 | Ref. | 21.6 | Ref. |
| 1-2 | 60.4 | 56.6 | 59.7 | 1.21 (0.67, 2.21) | 63.2 | 1.56 (0.90, 2.72) |
| ≥ 3 | 14.3 | 13.2 | 14.1 | 1.23 (0.57, 2.65) | 15.2 | 1.62 (0.81, 3.23) |

^a^ P values are based on multinomial logistic regression models for each socio-demographic, lifestyle and health characteristic in relation to Covid-19 level of concern, taking complex sampling design into consideration; ^b^ Column percentages are displayed for the distribution of socio-demographic, lifestyle and health characteristics, overall, and among individuals with medium, low and high levels of Covid-19 concern; ^c^ Relative risk ratio for ‘Low’ versus ‘Medium’ Covid-19 level of concern based on multinomial logistic regression model; ^d^ Relative risk ratio for ‘High’ versus ‘Medium’ Covid-19 level of concern based on multinomial logistic regression model. *Abbreviations:* AHEAD = Study of Asset and Health Dynamics of the Oldest Old; GED = General Educational Development; SEM = Standard error of the mean.

**Table S.2.** Super Learner model for predictors of Covid-19 level of concern – 2020 Health and Retirement Study enhanced interviewing Covid-19 half-sample

|  | **Covid-19 level of concern** | |
| --- | --- | --- |
|  | **‘Low’ vs. ‘Medium’** | **‘High’ vs. ‘Low’** |
| *Model A: LASSO* |  |  |
| cv-Risk | 0.247 | 0.221 |
| *Model B: Random Forest* |  |  |
| cv-Risk | 0.246 | 0.214 |
| *Model C: XGBOOST* |  |  |
| cv-Risk | 0.319 | 0.259 |
| *Model D: SVM* |  |  |
| cv-Risk | 0.250 | 0.229 |
| *Super Learner:* |  |  |
| cv-Risk | 0.247 | 0.213 |
| AUC | 0.485 | 0.664 |

*Abbreviations:* AUC = Area under the curve; cv = cross-validated; LASSO = Least Absolute Shrinkage and Selection Operator; SVM = Support Vector Machine.

**Table S.3.** Comparison of potentially eligible study participants (n=2,902) and non-participants (n=14,230) according to selected baseline characteristics – 2006-2020 Health and Retirement Study (n=17,132)

|  | **Study participants**  **(%)** | **Study**  **non-participants**  **(%)** | **P*** |
| --- | --- | --- | --- |
| ***Sex:*** |  |  | 0.58 |
| Male | 47.1 | 39.7 |  |
| Female | 52.9 | 60.3 |  |
| ***Birth cohort:*** |  |  | 0.25 |
| Original/AHEAD/Children of the Depression | 15.2 | 36.2 |  |
| War Babies | 14.8 | 0.30 |  |
| Early Baby Boomers | 20.3 | 13.1 |  |
| Mid Baby Boomers | 24.2 | 21.0 |  |
| Late Baby Boomers | 25.6 | 26.2 |  |
| ***Race:*** |  |  | 0.08 |
| White / Caucasian | 79.7 | 70.3 |  |
| Black / African American | 10.8 | 24.9 |  |
| Other | 9.5 | 0.5 |  |
| ***Ethnicity:*** |  |  | 0.03 |
| Hispanic | 10.0 | 25.4 |  |
| Non-Hispanic | 89.9 | 74.6 |  |
| ***Education:*** |  |  | 0.007 |
| No degree | 10.6 | 47.9 |  |
| GED | 5.4 | 0.0 |  |
| High school diploma | 25.2 | 16.9 |  |
| Some college | 28.0 | 7.8 |  |
| College degree or higher | 30.8 | 27.3 |  |

* P values were calculated based on design-based F tests.
